# Metabolomics for Biomarker Discovery: Moving to the Clinic

**DOI:** 10.1155/2015/354671

**Published:** 2015-05-19

**Authors:** Aihua Zhang, Hui Sun, Guangli Yan, Ping Wang, Xijun Wang

**Affiliations:** National TCM Key Lab of Serum Pharmacochemistry, Laboratory of Metabolomics and Chinmedomics, Department of Pharmaceutical Analysis, Heilongjiang University of Chinese Medicine, Heping Road 24, Harbin 150040, China

## Abstract

To improve the clinical course of diseases, more accurate diagnostic and assessment methods are required as early as possible. In order to achieve this, metabolomics offers new opportunities for biomarker discovery in complex diseases and may provide pathological understanding of diseases beyond traditional technologies. It is the systematic analysis of low-molecular-weight metabolites in biological samples and has become an important tool in clinical research and the diagnosis of human disease and has been applied to discovery and identification of the perturbed pathways. It provides a powerful approach to discover biomarkers in biological systems and offers a holistic approach with the promise to clinically enhance diagnostics. When carried out properly, it could provide insight into the understanding of the underlying mechanisms of diseases, help to identify patients at risk of disease, and predict the response to specific treatments. Currently, metabolomics has become an important tool in clinical research and the diagnosis of human disease and becomes a hot topic. This review will highlight the importance and benefit of metabolomics for identifying biomarkers that accurately screen potential biomarkers of diseases.

## 1. Introduction

Metabolomics, an omic science, deals with the global assessment of the metabolites present in a biological system to evaluate the progress of the disease, select potential biomarkers, and provide insights into the underlying pathophysiology [1]. Recent improvements in metabolomics technologies reveal the unequivocal value of metabolomics tools in biomarker discovery, gene-function analysis, systems biology, and diagnostic platforms. Because metabolites represent the downstream expression of genome, transcriptome, and proteome, they can closely reflect the phenotype of an organism at a specific time. Analyzing metabolic differences between unperturbed and perturbed pathways could provide insight into underlying disease pathology and disease prognosis and diagnosis. Additionally, small molecule metabolites can provide mechanistic insights into novel biomarkers for diseases, given the limitations of the current traditional markers [2]. Metabolomics may provide biomarkers useful for identification of early stage diseases, potentially addressing an important clinical need.

The development of metabolomic technologies holds the promise to significantly improve diagnosis, unravel more appropriate therapeutic targets, and enable more precise prognosis of disease development. The potential of this approach for clinical diagnostics is huge, since only minimal biological preparation is necessary. In recent years, the development of instrumental systems, such as high-resolution nuclear magnetic resonance spectroscopy (NMR) and mass spectrometry (MS), ultraperformance liquid chromatography, and more sophisticated bioinformatics and analytical techniques, has enabled more comprehensive coverage of the metabolome [3]. Advances in mass spectrometry coupled with chromatography have particularly improved both the efficiency and reliability of metabolic profiling and represent one of the major platforms in clinical fields [4]. Bioinformation derived from these techniques could provide accurate and clinically useful diagnostic capability for the management of diseases at the metabolic level in concentration [5]. With advantages of high throughput, high sensitivity, and high accuracy, metabolomics shows great potential and value in disease treatment.

Early detection is the most effective way to improve the clinical outcome of diseases. Emerging metabolomics provides a powerful platform for discovering novel biomarkers and biochemical pathways to improve early diagnosis.

Metabolites that are important indicators of physiological or pathological states can provide information for the identification of early biomarkers for disease and help to understand its progression [6]. Metabolomics has the potential to generate novel noninvasive diagnostic tests and provides a unique insight into established and novel metabolic pathways, which are simple and cost-effective yet retain high sensitivity and specificity characteristics [7]. Because of this potential, the objective of this paper is to find metabolites that can be used as a clinical test for the early diagnosis and monitoring of the disease and the outcome of therapy and discuss its significance in depth in the postgenomic era and it highlights clinical associations and potential challenges. Moreover, an overview of most interesting recent biomarker discovery publications is provided to highlight the clinical applications of metabolomic techniques used for bioanalytical data interpretation.

## 2. Metabolomics for Biomarker Discovery

Biomarkers of preclinical disease will be critical to the development of disease-modifying or even preventative therapies. The early detection of diseases is pivotal for successful patient treatment and management. With the recent emergence of new technologies, the biomarker discovery has been the subject of intense research and activity. The alterations of metabolites in biofluids are indicators of variations in physiology or pathology. Metabolomics is a well-established rapidly developing research field involving quantitative and qualitative metabolite assessment within biological systems [8–11]. The metabolome represents the endpoint of the omics cascade and is also the closest point in the cascade to the phenotype. Therefore, metabolome analysis can be a useful approach for finding effective diagnostic markers and examining unknown pathological conditions. Metabolomics involves the establishment of relationships between phenotype and a metabolic signature, which are key aspects of biological function, and is a powerful technique for the elucidation of biochemical pathways to improve diagnosis and therapy [12, 13]. It has the potential to provide insight into the pathogenesis of disease states and discover diagnostic markers for therapeutic targets. An advantage of this approach or the predictive power of metabolomics performed better in both sensitivity and specificity and may be of advantage in the future for the determination of biomarkers [14]. Moreover, metabolic profiling is highly simple, accurate, and specific and should prove equally valuable in metabolomic research applications.

## 3. Metabolomics in Diagnosis

Early diagnosis is the key to the effective treatment of disease. The detection of disease biomarkers plays a critical role not only in disease early diagnosis, but also in classification and disease progression or assessment prognosis and treatment response. Analysis of the key metabolites has become an important role to monitor the state of biological organisms. Metabolomics is an emerging analytical technique for systemic determination of metabolite profiles, which is useful for understanding the biochemical changes in related diseases [15]. It is increasingly being applied towards the identification of biomarkers for disease diagnosis and risk prediction [16]. Recent developments have suggested metabolomics is the relatively new field in bioinformatics that uses measurements on metabolite abundance as a tool for disease diagnosis and other medical purposes [17]. It holds promise for early diagnosis and increases choice of therapy and the identification of new metabolic pathways that could potentially be targeted in diseases [18]. Owing to the complexity and volume of the data generated by the advanced technologies in metabolomics, pattern recognition methods have become predominant in the medical sciences and may be suitable for certain diagnostics medical applications. It is hoped that the information derived from metabolite profiling will make it possible to suggest individualized therapies that more effectively treat disease. Improvements in analytical technologies have made it possible to rapidly determine the concentrations of thousands of metabolites in any biological sample, which has resulted in metabolome analysis being applied to various clinical studies. Advent of advanced metabolomics technologies and associated bioinformatics development is bringing these goals into focus. Metabolomics technology is still under development and is a promising tool for elucidating biological pathways and discovering clinical biomarkers, strengthening the efforts for optimizing both the prevention and treatment of disease.

## 4. Application in Disease Biomarker Discovery

Alzheimer's disease (AD) is a leading cause of morbidity and mortality and is a major epidemic worldwide. Unfortunately, current biomarkers for early disease are limited because they are either invasive, time-consuming, or expensive. A study combining high-resolution mass spectrometry and chemometrics for the analysis of AD was undertaken [19]. Predictive power of the metabolomics approach for distinguishing AD cases was confirmed, reaching 100% diagnostic accuracy. A nontargeted metabolomic approach was developed to examine metabolic differences subjects with different cognitive status related to AD progression [20]. Choline, dimethylarginine, arginine, valine, and so forth were identified as possible progression biomarkers. Their predictive values were higher than those reported with classical AD biomarkers. Blood-based biomarkers may be a more attractive option, but none can currently detect preclinical AD disease with the required sensitivity and specificity. Importantly, Mapstone et al. described our metabolomic approach to detecting preclinical AD disease in a group of cognitively normal older adults [21]. They discovered and validated a set of ten lipids from peripheral blood that predicted phenoconversion to either amnestic mild cognitive impairment or AD within a 2-3-year timeframe with over 90% accuracy. Such characteristic biomarkers will more accurately reflect AD pathophysiology, ultimately increasing the chances of success of preventive treatments.

Late diagnosis of hepatocarcinoma (HCC) is one of the most primary factors for the poor survival of patients. Thereby, identification of sensitive biomarkers for HCC early and accurate diagnosis is of great importance. In a study, Xiao et al. compared metabolite levels in sera of HCC and cirrhosis patients by metabolomics [22]. They verified the identities of selected putative identifications including glycolic acid and glycodeoxycholic acid. In a study done by Wang et al., thirteen potential biomarkers were identified and there were significant disturbances of key metabolic pathways, such as organic acids, phospholipids, fatty acids, bile acids, and gut flora metabolism in HCC patients [23]. In addition, glycochenodeoxycholic acid was suggested to be an important indicator for HCC diagnosis and disease prognosis. Metabolite signatures could be an efficient and convenient tool for early diagnosis and screening of HCC. Tan et al. used a nontargeted metabolomics method to find the potential biomarkers from the HCC disease model and test their usefulness in early human HCC diagnosis [24]. It was found that three marker metabolites (taurocholic acid, lysophosphoethanolamine 16 : 0, and lysophosphatidylcholine 22 : 5) achieved a sensitivity of 80.5% and a specificity of 80.1%, which are better than those of *α*-fetoprotein. Serum metabolites of the HCC patients and healthy controls were also investigated using the improved metabolomics [14]. 1-Methyladenosine that was identified as the characteristic metabolite for HCC exhibited significant improved sensitivity. Metabolite profiling of human HCC was performed by metabolomics in conjunction with multivariate statistical analyses [25]. Forty-three serum metabolites and 31 urinary metabolites were identified in HCC patients involving several key metabolic pathways such as bile acids, urea cycle, and methionine metabolism. These studies showed that metabolomics approach is a promising screening tool for the diagnosis of HCC patients. Shao and colleague developed a urinary pseudotargeted method based on LC-MS, which was applied to cirrhosis (CIR) and HCC investigation [26]. It was found that urinary nucleosides, bile acids, citric acid, and several amino acids were significantly changed in disease groups compared with the controls, featuring the dysregulation of purine metabolism, energy metabolism, and amino metabolism in liver diseases. Furthermore, butyrylcarnitine (carnitine C4:0) and hydantoin-5-propionic acid were defined as combinational markers to distinguish HCC from CIR. In a study, a novel steroid hormone metabolomic method based on liquid chromatography-mass spectrometry combined with logistic regression analysis was applied to investigate the steroid hormone disorders and to screen potential urinary steroid hormone biomarkers of early HCC [27]. Logistic regression analysis reveals that a panel of epitestosterone and allotetrahydrocortisol displayed excellent diagnostic capability for distinguishing early HCC. Determination of the metabolic alterations associated with the presence of HCC can improve our understanding of the pathophysiology of this cancer.

Traditional clinical biomarkers of renal function are not sensitive enough and only increase significantly after the presence of substantial chronic kidney diseases (CKD). Metabolomics approach was applied to establish a human CKD serum metabolic profile and the significant endogenous metabolites that contributed to distinguishing of CKD in different stages [28]. Based on these metabolites, the model for diagnosing patients with CKD achieved the sensitivity and specificity of 100% and may provide useful information for the early diagnosis of CKD. It illustrated that serum metabolic profile was altered in response to renal dysfunction and the progression of CKD. Several markers have been confirmed across multiple studies to detect CKD earlier than traditional clinical chemical and histopathological methods [29]. The application of metabolomics in CKD studies provides researchers with the opportunity to gain new insights into the pathophysiological mechanisms.

In search of new diagnostic biomarkers for ovarian endometriosis, Vouk et al. used a hypothesis-generating targeted metabolomics approach [30]. Eight lipid metabolites were identified as endometriosis-associated biomarkers. A model containing hydroxysphingomyelin 16 : 1 and the ratio between phosphatidylcholine 36 : 2 and ether-phospholipid 34 : 2 resulted in a sensitivity of 90.0% and a specificity of 84.3%. Results suggest that endometriosis is associated with elevated levels of sphingomyelins and phosphatidylcholines that may contribute to the suppression of apoptosis and affect lipid-associated signalling pathways. Metabolite profiling was used to select the metabolites to be used for the noninvasive diagnosis of liver-stagnation and spleen-deficiency syndrome- (LSS-) type disease [11]. Twelve urinary differential metabolites contributing to the complete separation of LSS patients from matched healthy controls were identified involving several key metabolic pathways such as pentose, cysteine, methionine, tyrosine, tryptophan, and nucleotide sugar metabolism. Importantly, 4 metabolite markers were effective for the diagnosis of human LSS, with an achieved sensitivity of 93.0%. Earlier detection of patients with metastatic colorectal cancer (mCRC) could improve their treatment and survival outcomes. In a study, Bertini et al. had used NMR to profile serum metabolome in patients with mCRC and determine whether a disease signature may exist that is strong enough to predict overall survival (OS) [31]. In the training set, NMR metabolomic profiling could discriminate patients with mCRC from healthy subjects with accuracy of 100%. In the validation set, 96.7% of subjects were correctly classified. A LC-MS based method has been carried out in conjunction with multivariate analysis to discriminate the global serum profiles of renal cell carcinoma patients [32]. As a result, 30 potential biomarkers for renal cell carcinoma are identified.

Jung et al. applied a NMR metabolomics approach to investigate the altered metabolic pattern in sera from patients with asthma and sought to identify the potential biomarkers [33]. Sera of asthma patients were characterized by increased levels of methionine, glutamine, and histidine and by decreased levels of formate, methanol, acetate, choline,* o*-phosphocholine, arginine, and glucose. In addition, potential biomarkers showed strong predictive power, and the presence of asthma in external validation models was predicted with high accuracy. Diagnostic and therapeutic biomarkers useful for esophageal squamous cell carcinoma (ESCC) have the ability to increase the long-term survival of cancer patients. In a pilot study, metabolomics study was originally carried out to determine global alterations in the metabolic profiles and find biomarkers potentially applicable to diagnosis [34]. Finally, 18 most significantly altered plasma metabolites in ESCC patients were tentatively identified as lysophosphatidylcholines, fatty acids,* L*-carnitine, acylcarnitines, organic acids, and a sterol metabolite. Moreover, variation of these three potential biomarkers was investigated over the treatment course and may be useful in diagnosing and monitoring therapeutic responses and predicting outcomes. In a study, Lam and coworker exploit metabolomic profiling for cancer biomarker discovery for diagnosis of malignant pleural effusions [35]. Insignificant markers were filtered out using a metabolome-wide significance level. The diagnostic performance of FFA 18 : 1-to-ceramide (d18 : 1/16 : 0) ratio supports use for diagnosis of malignant pleural effusions. Metabolomic profiling was performed using gas chromatography coupled to mass spectrometry, and forty-eight metabolites were identified in the serum samples [36]. Itaconic acid was found to be significantly more abundant in women who subsequently developed gestational diabetes mellitus and may have potential as a novel biomarker in early pregnancy to predict the subsequent development of gestational diabetes mellitus. These results demonstrate that robust metabolomics has the potential as a noninvasive strategy and promising screening tool to evaluate the potential of these metabolites in the early diagnosis of patients and provides new insight into pathophysiological mechanisms.

## 5. Current Trends and Future Perspectives

Many diseases result in characteristic changes in the metabolite profiles of fluids prior to development of clinical symptoms. These metabolites are often useful diagnostics biomarkers. Identifying biomarkers for the early detection of diseases will result in more efficient treatments, reduction in suffering, and lower mortality rates [37]. There is a dire need for sensitive and more affordable diagnostic tools to develop effective therapies. Besides the traditional techniques to investigate biomarker profiles, metabolomics appears to be an important tool to gain information on low-molecular-weight metabolites present in biofluids. Additionally, the high-throughput nature of metabolomics makes it ideal to perform biomarker screens for diseases or follow drug efficacy [38]. Metabolomics strategies to analyze, understand, and construct the metabolic pathways open the window of opportunity in a very cost-effective manner.

One of the major challenges in metabolomics is validation of fingerprint molecules to identify specifically perturbed pathways in metabolic aberrations. The identification and interpretation of metabolic biomarkers is also a challenging task. In this context, pathway-based approaches have increasingly become a key technology in metabolomics allowing capture of complex interactions in biological systems, with high-throughput evaluation of a large number of metabolites [39, 40]. While the past decade has seen great advancements in metabolomics research producing potential biomarkers, most of the identified biomarkers have failed to replace existing clinical tests. To become a clinically approved test, a potential biomarker should be confirmed and validated using hundreds of specimens and should be reproducible, specific, and sensitive. The quest for an ideal biomarker in disease is still underway. Since at present we cannot apply the application of the perfect marker, maybe combining different molecules will provide information compensating for the shortcoming of individual tests. The accumulated clinical research experience and the continuing exploration of the metabolomics ensure that there will be no shortage of newly discovered candidate biomarker molecules for the future. In the last decade, metabolomics has contributed substantially to our understanding of different diseases. The development of metabolome analysis will aid the discovery of novel biomarkers, hopefully leading to the early detection of various diseases [18, 41–43].

## 6. Conclusions

Metabolomics is an “omic” science that is now emerging with the purpose of elaborating a comprehensive analysis of the metabolome, which is the complete set of small molecules intermediates. Metabolome is a data-rich source of information concerning all the metabolites in a biofluid, which could indicate early biological changes to the host due to perturbations in disease. Metabolomics therefore has great potential for improving diagnosis, therapeutic treatment, and care of disease. In the past decade, metabolomics has already proved to be useful for the characterization of several pathological conditions and offers promises as a clinical tool. In future work, the potential biomarkers should be further validated with a large enough patient cohort to achieve earlier diagnosis. Moreover, the improvement of patient prognosis and care will be another aim of such investigations. In the era of personalized medicine with more and more patient specific targeted therapies being used, we need reliable and sensitive biomarkers to track the disease and to develop therapies during the course of treatment.
